# Labellar Structure of the *Maxillaria splendens* Alliance (Orchidaceae: Maxillariinae) Indicates Floral Polyphenols as a Reward for Stingless Bees

**DOI:** 10.3390/plants12040921

**Published:** 2023-02-17

**Authors:** Kevin L. Davies, Emerson R. Pansarin, Małgorzata Stpiczyńska

**Affiliations:** 1 School of Earth and Environmental Sciences, Cardiff University, Main Building, Park Place, Cardiff CF10 3AT, UK; 2 Department of Biology, FFCLRP, University of São Paulo, Av. Bandeirantes 3900, Ribeirão Preto 14040-901, São Paulo, Brazil; 3 Faculty of Biology, University of Warsaw, Botanic Garden, Al. Ujazdowskie 4, 00-478 Warszawa, Poland

**Keywords:** fluorescence microscopy, food-hair, food-reward, histochemistry, labellum, light microscopy, lipid, *Maxillaria*, Meliponini bees, papillae, pollination, polyphenols, protein, pseudopollen, resin, SEM, starch, trichomes

## Abstract

Several studies have reported stingless Meliponini bees gathering hairs from the labella of *Maxillaria* spp., including *M. ochroleuca*, a member of the *M. splendens* alliance. Such hairs usually contain food materials and are thought to have nutritional value. The papillose labella of representatives of the *Maxillaria splendens* alliance, however, bear scattered, simple 1-5-celled uniseriate trichomes (hairs) that lack food materials. By contrast, here, as well as polyphenolic compounds, typical labellar papillae usually contain small quantities of starch, protein, and minute droplets of lipid, the last probably involved in the production of fragrance. Towards the labellum apex occur elevated groups of papillae that lack food materials, but contain volatile compounds, probably fragrance precursors. In the past, the terms ‘trichomes’ or ‘hairs’ and ‘papillae’ have been used interchangeably, causing some confusion. Since the trichomes, however, unlike the papillae, are easily detachable and can fragment, it is most likely they, not the papillae, that have previously been observed being collected by bees, but their poor food content indicates that they do not function as food-hairs. Even so, our field observations of *M. ochroleuca* reveal that stingless bees scrape polyphenol-rich labellar tissue and possibly use this material to produce a resinous, complex, heterogeneous substance commonly referred to as ‘bee glue’, used for nest construction and repair.

## 1. Introduction

Although historically considered to be mainly rewardless, in recent years, a considerable number of species of *Maxillaria sensu lato* (*s.l.*) have been shown to produce floral rewards. These include nectar [1,2,3,4,5,6] and food-laden trichomes or papillae [7,8,9,10,11,12,13], in particular, pseudopollen or farina [3,5,7,8,9,14,15,16,17,18,19,20], a yellow-white, mealy material formed by the detachment or fragmentation of uniseriate, moniliform, multicellular, labellar trichomes to form individual or short chains of cells that are collected by pollinating stingless Meliponini bees [21,22]. Whereas the pseudopollen of *Maxillaria sensu stricto* (*s.s*.) usually contains starch and protein, these foods are sometimes absent, but by mimicking true pollen, it is thought that pseudopollen might still be able to attract insect pollinators by deceit [20,23]. The labella of some species of *Maxillaria s.l.* secrete heterogeneous, resin- or wax-like materials [3,4,5,7,8,9,11,24,25,26,27,28], and this is also gathered by Meliponini bees [11,29] and perhaps used to repair or line and waterproof the cells of their nests [16,30]. Yet, other closely related taxa, such as *Cyrtidiorchis* Rauschert [3] and those species of *Mormolyca* Fenzl that have insectiform flowers [3,13,31,32], and *Trigonidium* Lindl. [33], display sexual deceit.

Species of the *Maxillaria splendens* Poepp. & Endl. alliance, such as *M. buchtienii* Schltr., *M. chlorantha* Lindl., *M. ochroleuca* Lodd. ex Lindl. and *M. splendens* Poepp. & Endl., were assigned by Christenson [34] to sect. *Multiflorae* Christenson and are characterised by their caespitose habit, with strongly compressed ancipitous, unifoliate pseudobulbs subtended by several foliaceous bracts sub-similar to the leaves. These pseudobulbs often become ridged with age. Flowers are produced simultaneously in bosses arising from the axils of the uppermost foliaceous bracts and are arachniform with linear perianth lobes and three-lobed, usually pubescent labella, each bearing a simple oblong callus. They have a short column with an unadorned clinandrum and anther, though the latter may have a slightly thickened apex. The labella of all four of these representative species bear obpyriform papillae and identical simple, 3-4-celled, uniseriate trichomes [8,17,20,35]. Singer and Koehler [22] record that the labellar hairs of *M. ochroleuca*, *M. splendens* and *M. bradei* Schltr. ex Hoehne function as floral rewards. They observed *Trigona* bees collecting them from the tips of the labella of *M. ochroleuca* [8,22], but without pollinating the flowers, even though *Trigona* and other Meliponini bees are known to pollinate many species of *Maxillaria s.s*. and related genera [1,10,16,22,29,33,36,37]. This poses a conundrum since, although histochemistry has hitherto only been performed on the labellar trichomes and papillae of one closely related taxon, namely *M. buchtienii* [8,20], the trichomes of this species were shown to lack protein bodies, contain negligible amounts of starch, and no lipid, whereas greater quantities of the first two food materials were recorded for its labellar papillae [8,20]. The terms ‘hairs’, ‘trichomes’ and ‘papillae’ have been used interchangeably in the past and therefore, it is important to establish exactly what epidermal structures were collected. Since food substances in the labellar trichomes of members of the *M. splendens* alliance are seemingly scarce, in contrast to the labellar epidermal papillae, and since under low magnification the epidermal papillae towards the tip of the labellum appear almost farinaceous and arise in seemingly friable clusters, [20], we hypothesised that what Singer and Koehler [22] saw being gathered were in fact papillae, not trichomes [8]. Alternatively, if it were the labellar trichomes that were collected, the relative paucity of food materials they contain would indicate that it must be for resources other than foods, perhaps fragrances or other secretions useful to these insects, or even as material for lining their nests.

Here, we investigate the floral micromorphology, anatomy and histochemistry [38,39,40,41,42,43] of three representative species of the *Maxillaria splendens* alliance (abbreviations of author names follow Brummitt and Powell [44]), namely *Maxillaria ochroleuca* Lodd. ex Lindl., *M. pauciflora* Barb. Rodr. and *M. weberbaueri* Schltr., using light microscopy (LM), fluorescence microscopy (FM) and scanning electron-microscopy (SEM) for evidence of floral rewards, together with field observations of *M. ochroleuca* to understand the foraging behaviour of stingless bees and the pollination strategy employed. 

## 2. Results

### 2.1. Micromorphology

**Figure 1:**
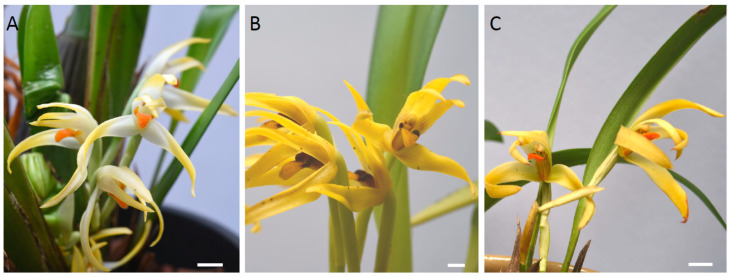
(**A**–**C**). Floral gross morphology of: (**A**). *Maxillaria ochroleuca*; (**B**). *Maxillaria pauciflora*; (**C**). *Maxillaria weberbaueri*. Scale bars = 5 mm.

The flowers of the three representative species of the *Maxillaria splendens* alliance investigated here, like those of *M. buchtienii* and *M. splendens* (K.L. Davies pers. obs. 1998, 2013), produced a characteristic ‘fruity’ fragrance. They had arachniform flowers with sepals and petals in various shades of yellow. The distinctly 3-lobed labella were linguiform and pubescent and contrasted strongly in colour with the other perianth segments, being either orange, as in *M. ochroleuca* and *M. weberbaueri*, or yellow with dark red lateral lobes as in *M. pauciflora* (Figure 1A–C). 

In all three species, the structure of the sepals and petals was similar, possessing a smooth epidermis. In *M. pauciflora*, the cells of the adaxial epidermis of the petals and sepals were convex/papillose. 

**Figure 2:**
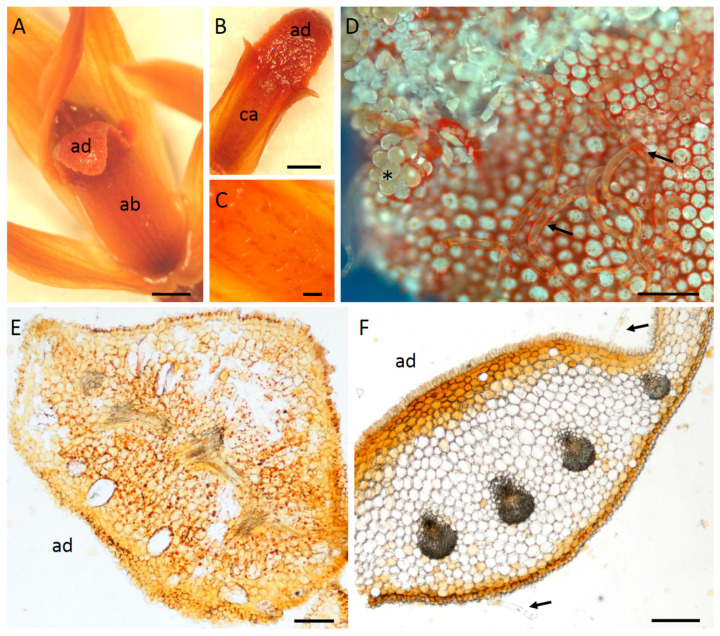
(**A**–**F**). Detail of flower of *M. ochroleuca* showing: (**A**). Reflexed apical part of labellum and both adaxial and abaxial surfaces. (**B**). Adaxial surface of labellum with elevated clusters of papillae towards its apex. (**C**). Detail of callus with scattered trichomes. (**D**). Elevated clusters of papillae (asterisk) and trichomes (arrows) on apical part of labellum, showing autofluorescence. (**E**). Hand-cut, unstained transverse section of the apical part of the labellum. Note the papillose adaxial and abaxial epidermis, and the large idioblasts (lacking raphides) within the parenchyma, also the abundant orange or red aggregates of perinuclear chromoplasts containing carotenoids. (**F**). Hand-cut, unstained transverse section through the callus showing regularly arranged papillose adaxial epidermal cells and typical abaxial epidermal cells. Both surfaces bear trichomes (arrows). Scale bars = 2 mm, 2 mm, 200 µm, 100 µm, 200 µm, 200 µm, respectively. ab = abaxial surface, ad = adaxial surface, ca = callus.

**Figure 3:**
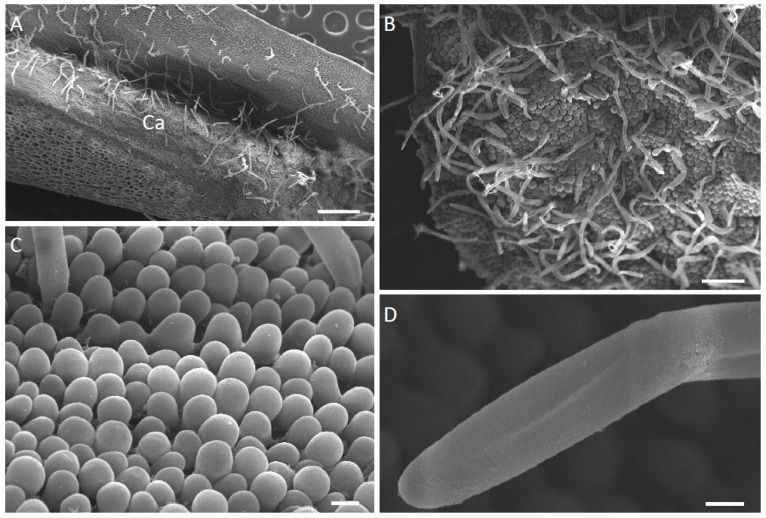
(**A**–**D**). Adaxial surface of the labellum of *M. ochroleuca*, SEM. (**A**). Callus and marginal region clothed with trichomes. (**B**). Apical part of labellum with few-celled trichomes and papillose epidermal cells. (**C**). Detail of epidermal papillae with smooth cuticle. (**D**). Detail of apical cell of a trichome. Note the seemingly intact cuticle and the absence of surface secretion. Scale bars = 500 µm, 150 µm, 20 µm, 10 µm, respectively. Ca = callus.

**Figure 4:**
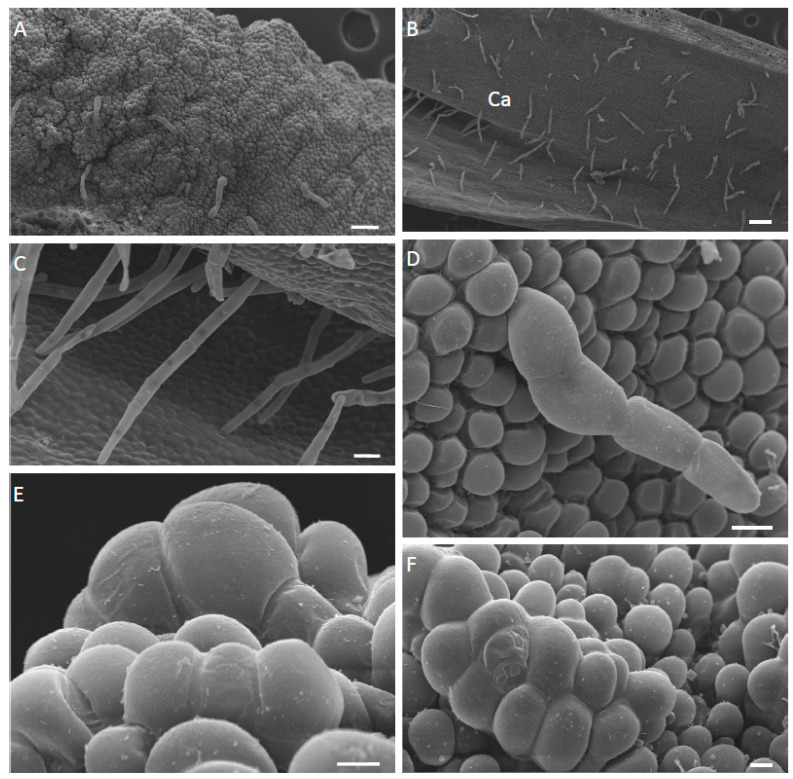
(**A**–**F**). Adaxial surface of the labellum of *M. pauciflora*, SEM. (**A**). Apical part of labellum bearing clusters of elevated papillae and few-celled trichomes. (**B**). Callus region with trichomes and typical epidermal cells. (**C**). Multicellular trichomes on callus and marginal part of labellum. (**D**). Multicellular trichome with smooth, seemingly intact cuticle, located on apical part of labellum. (**E**,**F**). Elevated papillae on apical part of labellum. Note that the smooth cuticle lacks traces of secretion. Scale bars = 100 µm, 200 µm, 30 µm, 20 µm, 10 µm, 10 µm, respectively. Ca = callus.

**Figure 5:**
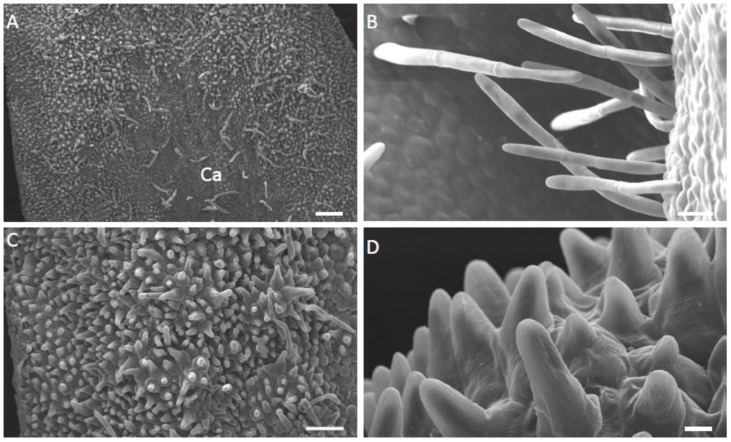
(**A**–**D**). Adaxial surface of the labellum of *M. weberbaueri*, SEM. (**A**). Central callus and marginal regions of labellum with scattered trichomes. (**B**). Multicellular, relatively long trichomes from the callus region. (**C**). Conical papillae and shorter trichomes from the apical part of the labellum. (**D**). Detail of conical papillae showing intact, smooth cuticle. Scale bars = 200 µm, 30 µm, 100 µm, 10 µm, respectively. Ca = callus.

**Figure 6:**
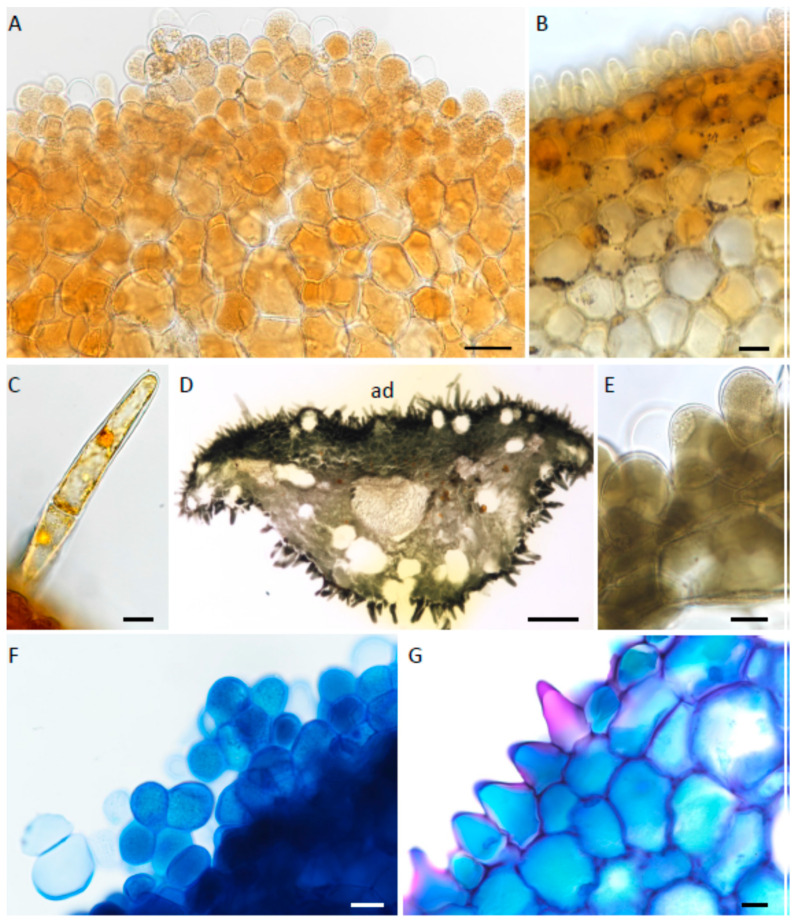
(**A**–**G**). Histochemistry of labellar tissue. (**A**–**C**). Labellar tissues treated with IKI for starch. (**A**). Epidermis and subepidermal parenchyma from apical part of labellum of *M. pauciflora* lacking obvious starch grains. (**B**). A similar preparation of *M. pauciflora*. Starch is present in the subepidermal parenchyma of the callus region. (**C**). Trichome of *M. pauciflora* lacking starch reserves. (**D**,**E**). Labellar tissues treated with FeCl_3_ for polyphenolic compounds. (**D**). Hand-cut, transverse section through the apical part of the labellum of *M. weberbaueri*. Note the intense staining of the epidermal papillae and subepidermal parenchyma. Large idioblasts are visible in the ground parenchyma. (**E**). Adaxial epidermal papillae and subepidermal parenchyma of *M. ochroleuca*. (**F**,**G**). Hand-cut, transverse sections of the apical part of the labella of *M. pauciflora* and *M. weberbaueri*, respectively, stained with TBO. Vacuoles of both epidermal papillae and subepidermal parenchyma cells stained blue-green. Scale bars = 50 µm, 20 µm, 15 µm, 200 µm, 20 µm, 20 µm, 20 µm, respectively. ad = adaxial surface.

The most obvious difference between these three species was the structure of their labella. In each case, the basal, median part of the labellum was adnate with the column-foot, and the apical part of the labellum reflexed (Figure 1A–C and Figure 2A). The outer tangential walls of the abaxial epidermal cells were either flat, as in *M. ochroleuca* (Figure 2E,F) and *M. pauciflora*, or papillose, as in *M. weberbaueri*. The simple, uniseriate, 1-5-celled trichomes had somewhat tapering or occasionally clavate, pointed to rounded terminal cells. Although they were generally scattered over the abaxial surface of the labellum, they were much more densely distributed over the entire adaxial surface of *M. pauciflora* and *M. ochroleuca*, (Figure 2B–D,F, Figure 3A–D and Figure 4A–D). In *M. weberbaueri*, they were present predominantly in the central callus area (Figure 5A,B). In *M. weberbaueri*, most of the unicellular, labellar epidermal papillae were conical (Figure 5A,C,D), particularly those at the labellar margins and apex, in contrast to *M. ochroleuca* (Figure 2D and Figure 3B,C) and *M. pauciflora* (Figure 4A,D–F), where they were obpyriform to spherical. Moreover, in all three species, *M. ochroleuca* (Figure 2D and Figure 3B), *M. pauciflora* (Figure 4A,E,F) and *M. weberbaueri* (Figure 5C), some of these papillae, whether conical or spherical, formed elevated multicellular clusters which stood proud of the adaxial labellar surface (Figure 4E,F and Figure 6A,F). The cuticle overlying both papillae and trichomes was smooth, intact and lacked obvious pores (Figure 3C,D, Figure 4C–F and Figure 5B,D). 

The ground parenchyma cells of the labellum, at anthesis, contained abundant chromoplasts enclosing yellow, orange or red carotenoids (Figure 2E), those of the sepals and petals being predominantly yellow. Comparative observations made on the labella of young flower buds and fully opened flowers revealed that these organelles were derived from leucoplasts, and at anthesis, formed perinuclear aggregates that obscured the profile of the nucleus. 

### 2.2. Histochemistry

In *M. pauciflora*, the adaxial subepidermal parenchyma cells of the sepals and petals contained significant quantities of starch (see Figures S1–S3). This was absent from abaxial tissues. Both the epidermis and parenchyma of these organs stained intensely for phenolic compounds with FeCl_3_. Scattered starch grains were also present in the parenchyma cells of *M. weberbaueri*. The petal and sepal cells of *M. ochroleuca* contained only small quantities of starch. However, *M. ochroleuca* differed from the other two species in that the cells of the adaxial epidermis and the subepidermal parenchyma of the sepals and petals contained numerous lipid droplets that stained with Sudan IV (Figures S1–S3) and fluoresced yellow-green after staining with NR.

**Figure 7:**
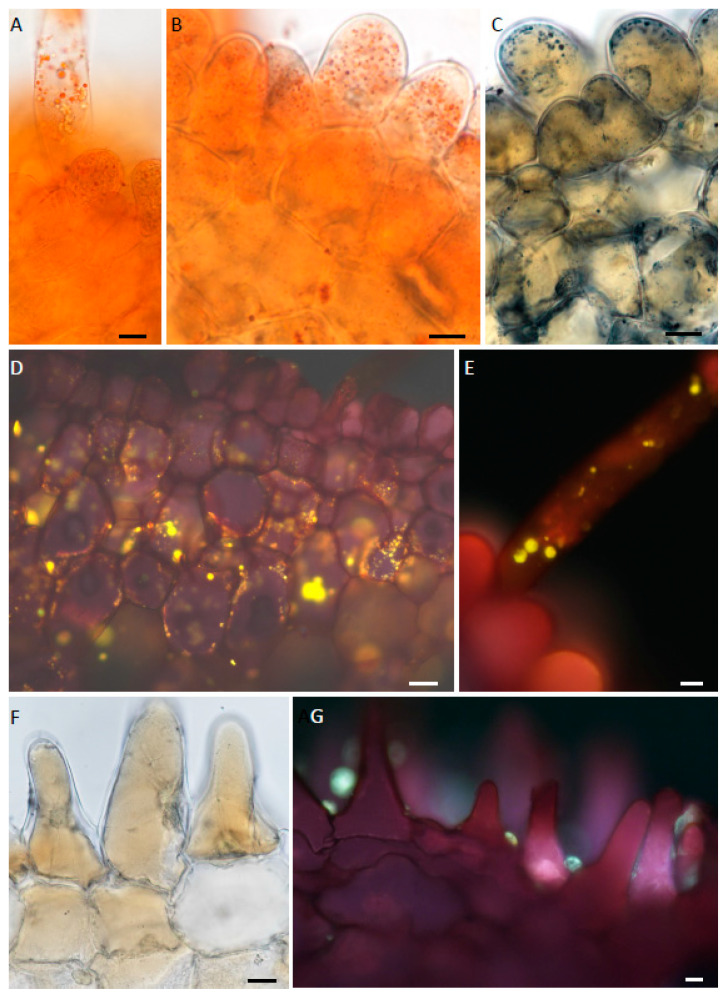
(**A**–**G**). Histochemistry of labellar tissue. (**A**,**B**). Labellar tissues treated with Sudan IV, and (**C**)., with SBB for lipids. (**A**). Lipid droplets in a trichome of *M. ochroleuca*. (**B**). Numerous minute lipid droplets in papillae of *M. pauciflora*. (**C**). Darkly stained lipid droplets in epidermal papillae and subepidermal cells of *M. ochroleuca*. (**D**,**E**,**G**). Labellar tissues stained with NR followed by fluorescence microscopy. (**D**,**E**). Lipid droplets in parenchyma cells and in a trichome, respectively, of *M. ochroleuca*, appear yellow-green. (**F**). Treatment with SBB did not detect lipids in the epidermal papillae of *M. weberbaueri*. (**G**). Surface secretion occurs between and upon the adaxial labellar papillae of *M. weberbaueri*. Scale bars = 10 µm, 20 µm, 20 µm, 20 µm, 10 µm, 20 µm, 50 µm, respectively.

The epidermal cells and subepidermal parenchyma of the labellum of each species contained a small amount of starch, mainly in the central callus region, but this was absent from trichomes (Figure 6A–C). No protein bodies were detected. The large idioblasts (Figure 2E,F and Figure 6D) scattered throughout the parenchyma contained bundles of raphides, but no mucilage. These raphides were easily displaced on section-cutting. In all investigated species, the vacuoles of epidermal papillae and subepidermal parenchyma had granular contents (Figure 6A) that stained intensely black with FeCl_3_ (Figure 6D,E) and blue-green with TBO (Figure 6F,G), indicating the presence of phenolic compounds. Staining with Sudan IV or SBB revealed for *M. ochroleuca* and *M. pauciflora* the presence of numerous, minute lipid droplets within epidermal papillae and trichomes located at the apical part of the labellum (Figure 7A–C). These were also visible by applying fluorescence microscopy to sections stained with NR (Figure 7D,E) and may indicate the presence of volatile compounds. Lipid droplets were absent from the labellar cells of *M. weberbaueri* (Figure 7F). Nevertheless, staining with NR revealed the presence of surface secretion between the epidermal papillae of this species (Figure 7G). Surface secretion, however, was not observed on the labella of *M. ochroleuca* or *M. pauciflora*. 

### 2.3. Field Observations and Bee Behaviour 

**Figure 8:**
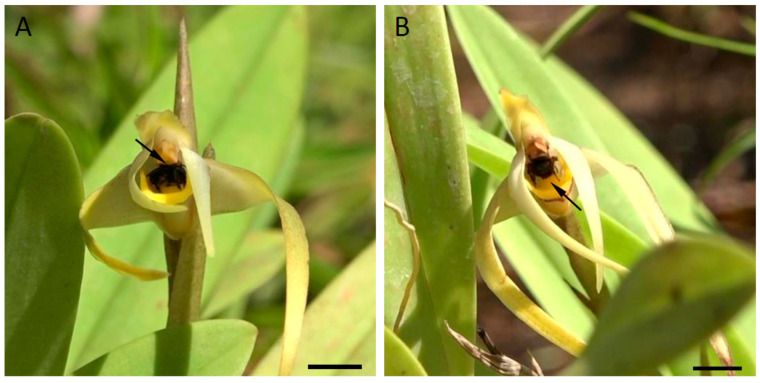
(**A**,**B**). Field observations. (**A**). The stingless bee *Trigona spinipes* entering a flower of *Maxillaria ochroleuca*. Note that the scutellum of the bee is in contact with the viscidium (arrow). (**B**). *Trigona spinipes* leaving a flower (anther cap and pollinarium removed). Note the callus (arrow) which elevates the body of the bee allowing contact between the dorsal surface of the insect thorax and the reproductive structures of the flower. Scale bars = 1 cm.

Workers of three species of Meliponini bee visited the flowers of *Maxillaria ochroleuca*, namely *Frieseomelitta varia, Scaptotrigona* aff. *depilis* and *Trigona spinipes* (Figure 8A,B). However, only workers of *T. spinipes* were observed transferring pollinaria between individual flowers of this species. Insect visits to flowers of *M. ochroleuca* were recorded between 08:30 and 13:10 h. Bees visited from one to four flowers per plant, and each visit lasted from a few seconds to two minutes. Visits commenced with bees alighting on the sepals, petals, or directly on the labellum. They subsequently wandered over the surface of these structures before entering headlong the gap between the column and labellum and scraping with their forelegs the papillae adjacent to the callus and at the base of the labellum (Video S1). The collected labellar material was transferred to the ventral surface of the thorax and eventually, to the corbiculae on the hind legs. As the bee passed beneath the anther, the pollinarium was deposited on the scutellum. A few seconds later, the anther-cap became detached and was shed. On visiting another flower, at least one of the four pollinia found in each pollinarium made contact with the stigma. The role of the labellar callus, which is located beneath the reproductive structures of the flower, is to elevate the bee so that its scutellum and the pollinarium it bears come into contact with the reproductive organs of the flowers, thus facilitating pollination.

## 3. Discussion

Our labellar morphology data presented here for *M. ochroleuca* and *M. pauciflora* are similar to those previously published for members of the *M. splendens* alliance such as *M. ochroleuca* [22,35], *M. splendens* [17], *M. chlorantha* [8] and *M. buchtienii* [8,20]. The labella comprise obpyriform to globose or spherical, unicellular papillae and scattered, simple, uniseriate 1–4-celled trichomes. Rarely, 5-celled trichomes occur. All these trichomes have somewhat tapering, pointed, rounded or clavate apices, a characteristic combination which appears to be constant for this alliance [8,35]. The labellar micromorphology of *M. weberbaueri* was also very similar except that the papillae were mainly conical. 

Elevated clusters of papillae also occurred on the labella of all three species, mainly towards the apex, giving it a somewhat farinaceous appearance. Similar configurations have previously been observed and illustrated for other members of Maxillariinae [17,20,35]. 

Generally, the labellar papillae of all species contained small amounts of starch and no obvious protein bodies or lipid droplets. Their intravacuolar contents, however, stained for polyphenols. Labellar trichomes generally lacked starch, protein, and lipid bodies. However, trichomes and papillae located at the apex of the labellum of *M. ochroleuca* and *M. pauciflora* contained numerous minute lipid droplets, but these were not visible in comparable cells of *M. weberbaueri.* These results are in broad agreement with those obtained for *M. buchtienii* in that the labellar trichomes of that species lacked protein, starch and lipid, and labellar papillae contained starch and protein, but no lipid [8]. Further histochemical testing of the labellum of *M. buchtienii* [20] revealed that the papillae contained small amounts of starch, protein and polyphenols, but no lipid and that some of the trichomes contained minute amounts of starch, stained for protein, but again, lacked lipid. Discrepancy in protein results between the two previous studies when compared with the present investigation can be explained in terms of the histochemical test employed. In the earlier studies [8,20], the xanthoproteic test was used to test for aromatic amino acids, and hence protein, whereas in the present investigation, Coomassie Brilliant Blue R250 was employed. Unfortunately, there is no satisfactory specific histochemical test for protein, and the xanthoproteic test, as well as being widely used for the detection of aromatic amino acids, may also detect other compounds with an activated benzene ring (see [20] and references therein). This nitroso reaction resulted in cytoplasmic staining, presumably due to detectable levels of cytoplasmic aromatic amino acid-containing proteins which stain orange. In the case of Coomassie Brilliant Blue R250, proteins bind to the reddish stain in acid solution and their positive charges suppress protonation resulting in a blue reaction product. Therefore, cytoplasm subjected to the xanthoproteic test stained more intensely for protein than when treated with Coomassie Brilliant Blue R250 and thus gave a more obviously positive result. Regardless of the stain used, only diffusely distributed cytoplasmic proteins, rather than discrete protein bodies, were detected. 

Labellar surface secretion of unknown chemical composition was present between the papillae and trichomes of *M. weberbaueri*, but not those of *M. ochroleuca* and *M. pauciflora.* Nevertheless, similar secretory material was observed in *M. buchtienii*, and it has been speculated that the blistering of the cuticle of labellar papillae in this species might be due to the discharge of fragrance [20]. Although all three investigated species are fragrant, in all cases, the thin cuticle overlying the labellar papillae and trichomes lacked evidence of blistering or of pores, indicating that fragrance mainly diffuses through the intact cuticle. 

Elevated clusters of papillae contained small lipid droplets. Staining with NR and examining under UV light indicated that these droplets were composed of volatile compounds. Such elevated clusters of papillae have been observed on the adaxial labellar surface of other *Maxillaria* species and related genera that, previous to recent revision [3], had once been assigned to that genus (e.g., *Camaridium meleagris*, Figure 3D [8]; *Camaridium obscurum* Figure 10C [27]; *Maxillariella ponerantha*, Figure 5E [35]; and *Christensonella mosenii* Figure 9I [35]). Those papillae that occurred towards the labellar apex of representative species of the *Maxillaria splendens* alliance appeared somewhat powdery under low magnification. Singer and co-workers [22] and R.B. Singer (pers. comm. in [8]) observed *Trigona* bees gathering ‘floral trichomes’ from the tips of the labella of *M. ochroleuca*, thus it might be that it is these papillae, not actual hairs, that were collected. This, however, does not preclude the possibility that true trichomes were also collected, but since the latter generally lack food reserves, containing only small droplets of oil or volatile compounds, if this is the case it would probably be for other resources, such as for fragrances that they might contain, or as material for nest building. 

Friable, epidermal farinaceous structures that in some ways resemble these elevated clusters of papillae can be found in *Dendrobium unicum* [45] and are referred to as granulae, comprising 3–12 cells that fall apart as individual or blocks of four cells or ‘tetrads’. These authors described how granulae soon disappeared from the labellar surface of *D. unicum*, and they suspected that they had been collected by small bees and wasps. Davies and Turner [46] reported that these granulae appear to be multicellular trichomes, comprising a stalk cell bearing a multicellular head of eight or nine cells, but in their study, instead of separating into ‘tetrads’, they fragmented to form individual or small groups of cells. Kjellsson and Rasmussen [45] interpreted these structures to be ‘food-hairs’, but did not test them for nutritional value, comparing them with pseudopollen on the basis that they are farinaceous, fragment and were removed, together with pollinaria, by pollinators, and asserting that pseudopollen need not necessarily contain food reserves [23]. Since then, however, Davies and Turner [46] have demonstrated histochemically that each individual cell of the trichome head of *D. unicum* contains starch, and on testing with the xanthoproteic test, showed that the cytoplasm contained relatively low concentrations of aromatic amino acids. Histochemistry did not detect the presence of lipid, but TEM demonstrated that small, scattered, lipid droplets were indeed present in the cytoplasm, together with mitochondria and plastids, the latter containing plastoglobuli but few lamellae. Based on their results, as well as field work by Kjellsson and Rasmussen [45], Davies and Turner [46] also arrived at the conclusion that these granulae had the potential to function as pseudopollen. The elevated clusters of papillae present at the labellum apex of members of the *M. splendens* alliance, however, despite superficial similarities, clearly differ from the food-hairs of *D. unicum* in that they are not easily detachable, are not friable and generally lack food reserves, containing numerous minute droplets of lipid only, interpreted here as droplets of volatile compounds. We therefore conclude that any insect attraction to these structures is due to their fragrance and possibly their configuration. 

Similarly, the labellar trichomes of this alliance also generally lack food reserves except for occasional small lipid droplets, possibly the precursors of fragrance production. However, unlike the clusters of labellar papillae, these trichomes are delicate, easily detachable and fragment, and as such, could easily be gathered by visiting insects. It is likely, therefore, that it is these that Singer and co-workers observed being collected by Meliponini bees from the labella of *M. ochroleuca* [22], [R.B. Singer pers. comm. in Davies and Turner, [8]], and if so, it was probably for the volatiles that they contain or for nest-building. 

Furthermore, based on our field observations, Meliponini bees actively scrape at the papillae adjacent to the callus and at the base of the lip. Histochemical tests revealed that these papillae are rich in phenolic compounds. Polyphenols are common constituents of both the resins collected by bees from plants and the ‘bee-glue’ produced in their nest [47]. Many of the properties of both these materials have been investigated [47,48,49,50,51]. Nevertheless, both substances have inconsistently been referred to as propolis or cerumen [48,51]. Propolis (bee-glue) is a complex and heterogeneous resinous mixture made by bees from collected plant-derived and other substances. These substances include polyphenols or resin, insect saliva, beeswax and exudate from a variety of vegetative botanical sources (e.g., tree buds and sap)., and are used by bees for constructing and repairing their nests [48]. Thus, its exact chemical composition depends on the prevailing environmental conditions and botanical material locally available, and it possesses both antioxidant and antibacterial properties [49,50]. It is widely stated that only two plant genera are known to produce floral resin used in the production of propolis, namely *Clusia* (Clusiaceae) and *Dalechampia* (Euphorbiaceae). However, it has long been proposed that floral resins and related compounds are also collected by Meliponini bees, also presumably for nest building, from a number of orchid genera. Many of these, such as *Heterotaxis*, *Rhetinantha*, and indeed, some species of *Maxillaria s.s.* [11,24,26], were formerly assigned to *Maxillaria s.l.*, and can now, presumably, also be added to their ranks. Furthermore, it is possible that some of these species may not be restricted to producing a single type of floral reward since *Maxillaria dichroma* produces both abundant, sunken, resiniferous floral trichomes, as well as pseudopollen produced by the fragmentation of multicellular, uniseriate, moniliform trichomes [25]. That, however, is another story. Sufficient to say here that Meliponini bees attracted to flowers of *M. ochroleuca* by volatile fragrant compounds are rewarded with polyphenol-rich material that may function as precursors for the production of a resinous substance used in the construction and repair of their nests. 

## 4. Materials and Methods

### 4.1. Plant Material

Three representatives of the *Maxillaria splendens* Poepp. & Endl. alliance, namely *Maxillaria ochroleuca* Lodd. ex Lindl. (Accession No. KLD 201719), *M. pauciflora* Barb. Rodr. (Accession No. KLD 201842) and *M. weberbaueri* Schltr. (Accession No. KLD 201929) were obtained from the first author’s collection and voucher specimens deposited at the Royal Botanic Gardens, Kew under the general Kew accession number Davies 2023 1, and at the Botanic Garden of the University of Warsaw under accession numbers 002671, 002672 and 002673, respectively. Author names follow Brummitt and Powell [44].

### 4.2. Location of Sites of Fragrance Production

For each species, secretory tissues representing putative sites of fragrance production were located by submerging entire flowers in an aqueous solution of 0.05% (*w*/*v*) Neutral Red (NR) for 20 min [38] and the distribution of stain recorded was compared with untreated control flowers. 

### 4.3. Histochemistry and Light Microscopy (LM)

A Nikon Eclipse Ni-U microscope with Nomarski optics was used for conventional, bright-field, light microscopy (LM) and Nomarski differential interference microscopy (NDIM) of putative fragrance-producing floral secretory tissues. For general histology, tissue samples were fixed in 2.5% (*v*/*v*) glutaraldehyde/4% (*v*/*v*) formaldehyde in phosphate buffer pH 7.4 and hand-cut sections were stained with 0.05% (*w*/*v*) aqueous Toluidine Blue O (TBO) solution [39]. 

Sections were also tested for lipids using a saturated ethanolic solution of Sudan IV or with Sudan Black B (SBB) where the natural orange colouration of cells obscured staining with the former; for starch using IKI (aqueous solution of iodine–potassium iodide); for mucilage using ruthenium red (RR) and for proteins using Coomassie Brilliant Blue R250 [40,41]. A 10% (*w*/*v*) aqueous solution of FeCl_3_ was used to test for the presence of phenolic compounds [42]. Control sections were used in each case. Fluorescence microscopy (FM) was undertaken using the above equipment in conjunction with a Prior 200 w lamp (Prior Scientific Instruments Ltd.) with a UV-2B cube filter (330–380 nm excitation filter; a 400 nm (LP) dichroic mirror and a 435 nm (LP) barrier filter). Intrinsic autofluorescence of hand-sectioned material was employed to reveal the presence and type of plastids, cuticle, cell walls, resins and wax. Sections treated with NR were examined under UV light for the presence of volatile oils [43]. Micrometry and photomicrography were undertaken using a DS-Fi2 high-definition colour digital camera and NIS-Elements imaging software (Nikon). 

### 4.4. Scanning Electron Microscopy (SEM) 

Tissue samples fixed as above were dehydrated in acetone, subjected to critical-point drying using liquid CO_2_, sputter-coated with gold and examined by means of a LEO 1430VP (Zeiss) scanning electron microscope (SEM) at an accelerating voltage of 30 kV.

### 4.5. Field Observations and Bee Behaviour 

Field studies were confined to the pollination of *Maxillaria ochroleuca* and the foraging behaviour of bees visiting its flowers. Observations were carried out in Ribeirão Preto municipality in southeastern Brazil during the periods 6–11 August and 1–6 September, 2020. Each daily period of observations lasted from 08:00 to 16:00 h, totalling 80 h. Insect visitors to flowers were recorded using a Sony FDR-AX100 4k Handycam.

Insects visiting these flowers were collected, identified and deposited at the “Pollinator Collection” of the LBMBP Laboratory, Department of Biology, Faculty of Philosophy, Sciences and Literature of Ribeirão Preto, University of São Paulo, Brazil.

## Data Availability

No new data were created.

## Supplementary Materials

The following supporting information can be downloaded at: https://www.mdpi.com/article/10.3390/plants12040921/s1. Figure S1: *Maxillaria ochroleuca*, hand-sections of sepals (A,B) and petals (C,D). A,C—sections stained with Sudan IV for lipids. B,D—sections stained with IKI; Figure S2: *Maxillaria pauciflora*, hand-sections of sepals (A,B) and petals (C,D). A,C—sections stained with Sudan IV for lipids. B,D—sections stained with IKI; Figure S3: *Maxillaria weberbaueri*, hand-sections of sepals (A,B) and petals (C,D). A,C—sections stained with Sudan IV for lipids. B,D—sections stained with IKI; Video S1: Reward collection by stingless bees on flowers of *Maxillaria ochroleuca*.
