# Supplementary figures and images for: Labellar Structure of the Maxillaria splendens Alliance (Orchidaceae: Maxillariinae) Indicates Floral Polyphenols as a Reward for Stingless Bees
Source: Plants (Basel). 2023 Feb 17;12(4):921. doi: 10.3390/plants12040921 (PMC9964541; doi:10.3390/plants12040921)

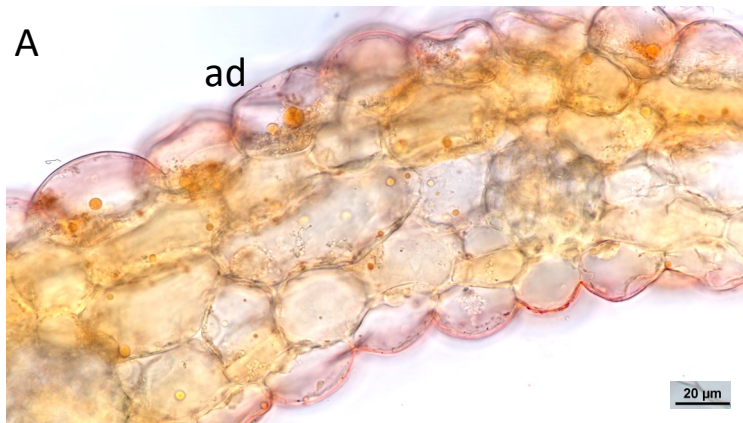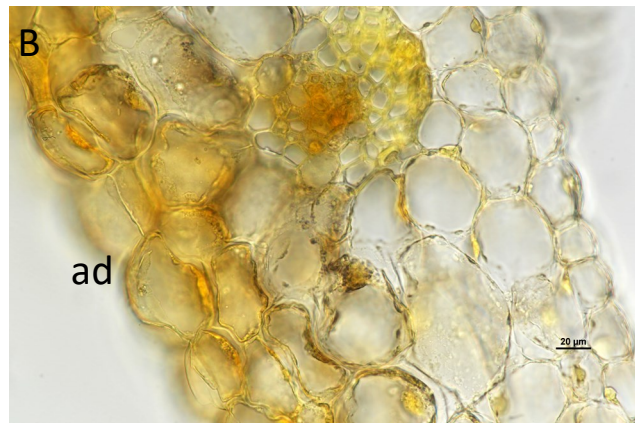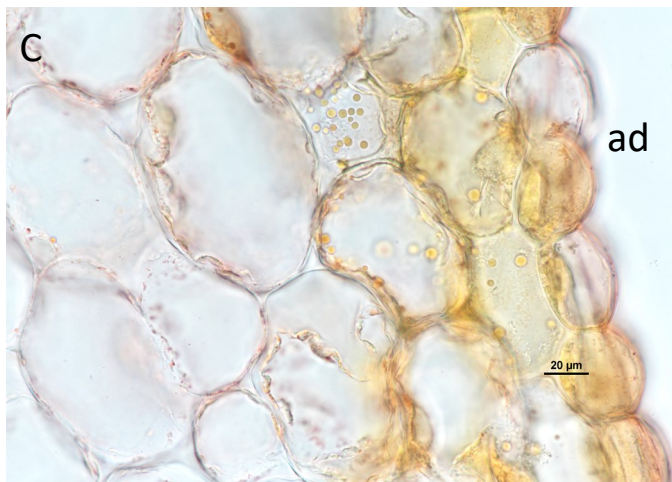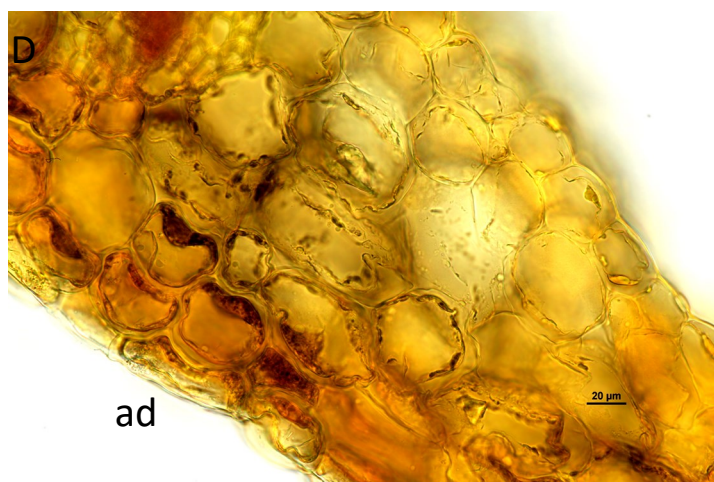

Supplement: Supplementary file 1 [file plants-12-00921-s001.zip › Figure S1. M. ochroleuca. Sepals and petals.pdf]

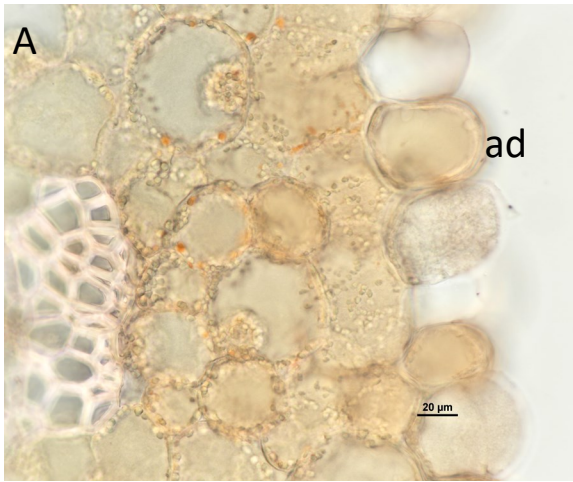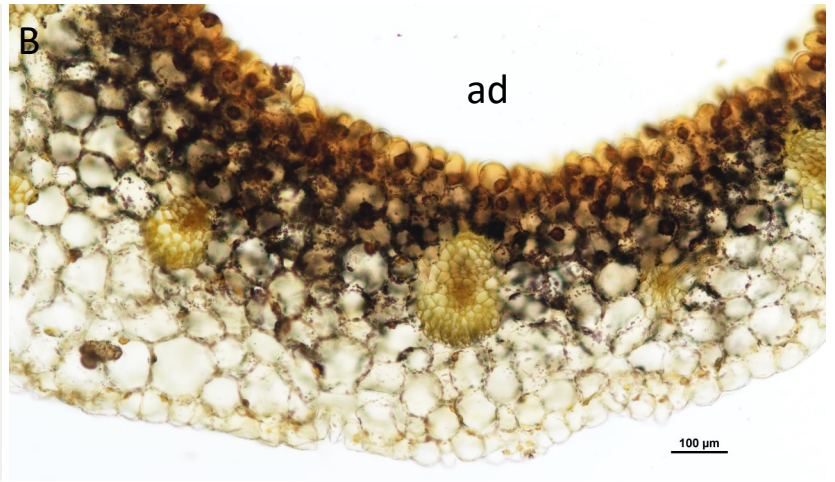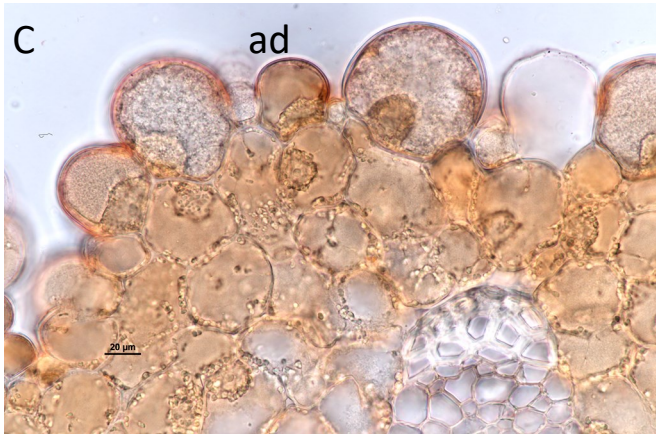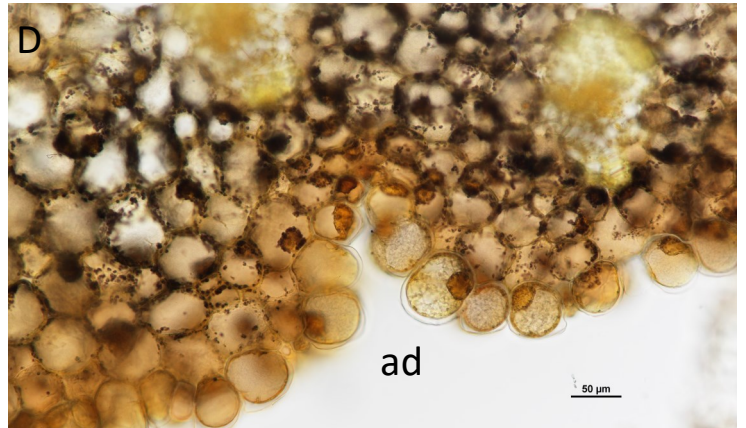

Supplement: Supplementary file 1 [file plants-12-00921-s001.zip › Figure S2. M. pauciflora. Sepals and petals.pdf]

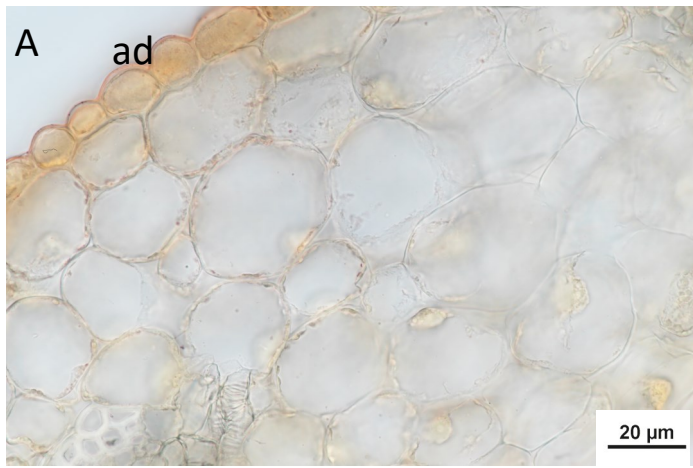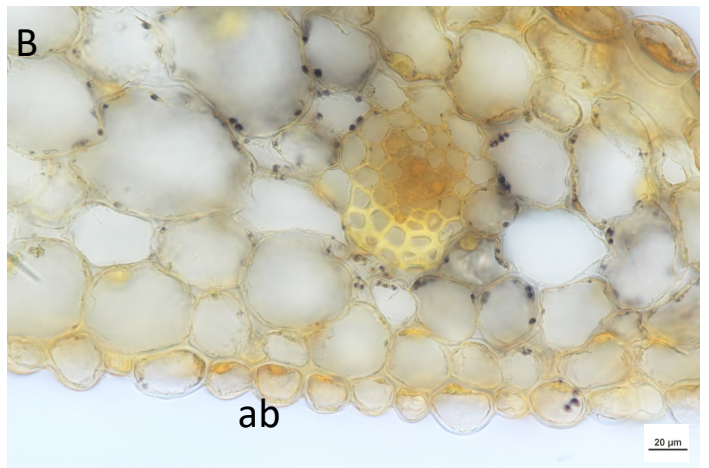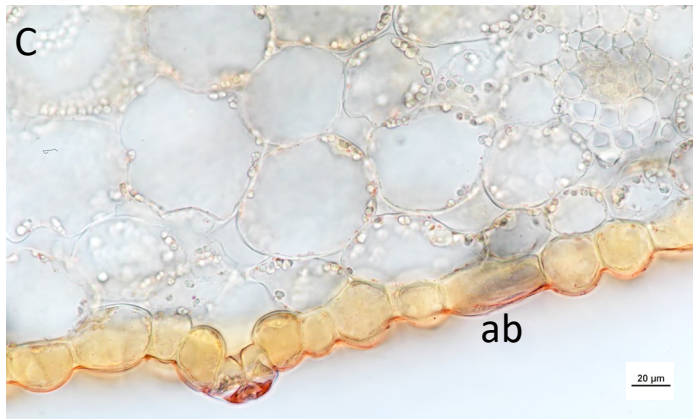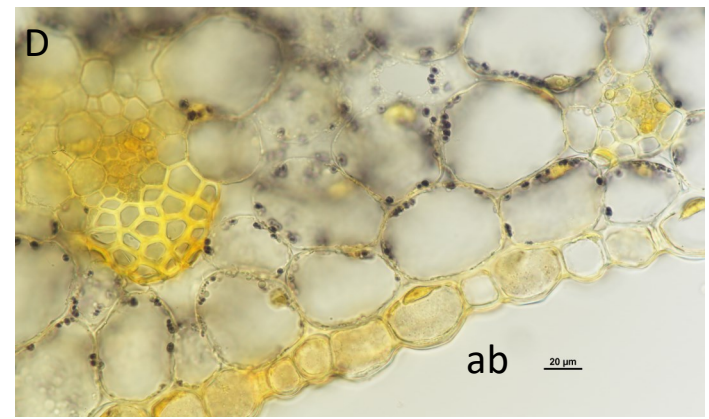

Supplement: Supplementary file 1 [file plants-12-00921-s001.zip › Figure S3. M. weberbaueri. Sepals and petals.pdf]
